# Macrophage-like THP-1 Cells Derived from High-Density Cell Culture Are Resistant to TRAIL-Induced Cell Death via Down-Regulation of Death-Receptors DR4 and DR5

**DOI:** 10.3390/biom12020150

**Published:** 2022-01-18

**Authors:** Yana Vladimirovna Lomovskaya, Margarita Igorevna Kobyakova, Anatoly Sergeevich Senotov, Alexey Igorevich Lomovsky, Vladislav Valentinovich Minaychev, Irina Sergeevna Fadeeva, Daria Yuryevna Shtatnova, Kirill Sergeevich Krasnov, Alena Igorevna Zvyagina, Vladimir Semenovich Akatov, Roman Sergeevich Fadeev

**Affiliations:** 1 Institute of Theoretical and Experimental Biophysics, Russian Academy of Sciences, 142290 Pushchino, Russia; yannalomovskaya@gmail.com (Y.V.L.); ritaaaaa49@gmail.com (M.I.K.); a.s.senotov@gmail.com (A.S.S.); lomovskyalex@gmail.com (A.I.L.); vminaychev@gmail.com (V.V.M.); aurin.fad@gmail.com (I.S.F.); shtatnovady@gmail.com (D.Y.S.); kirill.krasnov64@gmail.com (K.S.K.); alennazvyagina@gmail.com (A.I.Z.); vladimir.akatov@gmail.com (V.S.A.); 2 Pushchino State Institute of Natural Science, 142290 Pushchino, Russia

**Keywords:** leukemia cells, high-density culture, TRAIL-induced cell death, TRAIL receptors, macrophage-like phenotype, cell proliferation

## Abstract

**Simple Summary:**

The mechanisms of leukemic cell resistance to antitumor immunity remains a topical issue. In this work, we found an increase in TRAIL-resistance of human acute myeloid leukemia cells THP-1 in high-density populations in vitro. The results obtained show that a macrophage-like phenotype of the acute myeloid leukemia cells, caused by stressful conditions in high-density culture, can increaser resistance to TRAIL-induced apoptosis, while retaining proliferative potential. The mechanism of the increase in TRAIL-resistance can be related to a decrease in the expression of death receptors DR4 and DR5. The possible realization of these events in vivo may be the reason for tumor progression.

**Abstract:**

Tumor necrosis factor-related apoptosis-inducing ligand (TRAIL/Apo2L) is a highly selective and promising anticancer agent due to its specific apoptosis-inducing effect on tumor cells, rather than most normal cells. TRAIL is currently under investigation for use in the treatment of leukemia. However, the resistance of leukemic cells to TRAIL-induced apoptosis may limit its efficacy. The mechanisms of leukemic cell resistance to antitumor immunity remains a topical issue. In this work, we have found an increase in the resistance to TRAIL-induced cell death in human leukemia THP-1 cells, which was caused by differentiation into a macrophage-like phenotype in high-density culture in vitro. Stressful conditions, manifested by the inhibition of cell growth and the activation of cell death in high-density culture of THP-1 cells, induced the appearance of cells adhered to culture dishes. The THP-1ad cell line was derived by selection of these adhered cells. The genetic study, using STR and aCGH assays, has shown that THP-1ad cells were derived from THP-1 cells due to mutagenesis. The THP-1ad cells possessed high proliferative potential and a macrophage-like immunophenotype. The adhesion of THP-1ad cells to the extracellular matrix was mediated by αVβ5 integrin. The cytokine production, as well as the rise of intracellular ROS and NO activities by LPS in THP-1ad cell culture, were characteristic of macrophage-like cells. The THP-1ad cells were found to appear to increase in resistance to TRAIL-induced cell death in comparison with THP-1 cells. The mechanism of the increase in TRAIL-resistance can be related to a decrease in the expression of death receptors DR4 and DR5 on the THP-1ad cells. Thus, the macrophage-like phenotype formation with the maintenance of a high proliferative potential of leukemic cells, caused by stress conditions in high-density cell cultures in vitro, can induce an increase in resistance to TRAIL-induced cell death due to the loss of DR4 and DR5 receptors. The possible realization of these events in vivo may be the reason for tumor progression.

## 1. Introduction

TNFα-related apoptosis inducing ligand (TRAIL) is one of the key effectors of antitumor immunity, and the increase in the resistance of leukemic cells to TRAIL-induced apoptosis is an important mechanism required to overcome the antitumor control of the immune system [1,2]. The acquisition of resistance of leukemic cells to TRAIL-induced apoptosis can be mediated both by the suppression of intracellular TRAIL-dependent proapoptotic signaling pathways [3] and by the impaired expression of TRAIL receptors on the cell surface, including the loss of receptors DR4 and DR5, or an increase in the number of receptors DcR1 and DcR2 [4,5,6,7]. The modulation of cell sensitivity to TRAIL appears to have an important role in the regulation of hematopoiesis [8,9]. The induction of granulocytic differentiation in promyelocytic leukemia HL-60 cells by dimethyl sulfoxide (DMSO) can be accompanied by an increase in their TRAIL-resistance [10]. Apparently, the appearance of the signs of differentiation in leukemic cells induced by exogenic stimuli is consistent with the formation of resistance to TRAIL-mediated apoptosis within them [11]. The human leukemia THP-1 cell line is a well-known in vitro cell model for the immune modulation approach. THP-1 cells can be differentiated in vitro into a macrophage-like phenotype using PMA or other stimuli [12]. At the same time, the spontaneous appearance of macrophage-like cells was reported in populations of THP-1 without the action of any exogenous inducers [13,14,15]. However, there are no data on the TRAIL-resistance of these differentiated THP-1 cells. This work shows that the appearance of THP-1 cells, attached to the extracellular matrix in high-density culture under stress conditions, was manifested by the suppression of cell proliferation and the activation of cell death. Cell line THP-1ad was derived by using this population of attached cells. It was shown that THP-1ad cells developed due to mutagenesis in the THP-1 cells, which led to the appearance of macrophage-like features in them and increased resistance to TRAIL-induced cell death, while maintaining a high proliferative potential. The mechanism of the increase in resistance to TRAIL-induced cell death can be related to the loss of receptors DR4 and DR5 on the THP-1ad cells. The possible realization of this differentiation in vivo can increase the protection of acute myeloid leukemia cells against the immune system and enhance the potential for tumor progression.

## 2. Materials and Methods

### 2.1. Chemicals

MitoTracker Green FM, LysoTracker Green DND-26, pHrodo Green *E. coli*, DAF-FMDA, and SYBER Green were purchased from Thermo Scientific (Waltham, MA, USA). Fetal bovine serum was from Gibco (Gibco, Sigma-Aldrich Company Ltd., Waltham, MA, USA). Intracellular staining permeabilization wash buffer, MojoSort Human Pan Monocyte Isolation Kit, Human TruStain FcX (Fc receptor blocking solution), PE anti-human Ki-67, isotype control antibodies PE Mouse IgG1 k isotype Ctrl, APC Mouse IgG1 k isotype Ctrl, FITC Mouse IgG1 k isotype Ctrl, PE Mouse IgG2a k isotype, and antibodies APC anti-human CD11b, FITC anti-human CD11a, PE anti-human CD284, PE anti-human CD36, PE anti-human CD33, PE anti-human HLA-DR, PE anti-human CD262 (DR5,TRAIL-R2), APC anti-human CD261 (DR4, TRAIL-R1), and PE anti-human CD264 (DcR2, TRAIL-R4) were from BioLegend (San Diego, CA, USA). MycoFluor™ mycoplasma detection kit was from Molecular Probes Inc. (Eugene, OR, USA). FITC Mouse anti-human CD163, FITC anti-human CD68, and Alexa Fluor 647 Mouse anti-human CD263 (DcR1, TRAIL-R3) were from BD Bioscience (Franklin Lakes, NJ, USA). Culture media DMEM, F12 and RPMI 1640, 2-mercaptoethanol, solution of trypsin (0.025%), and EDTA (0.01%) in PBS, Calcein AM, bisbenzimide Hoechst 33342 (H-33342), 2′,7′-dichlorofluorescin diacetate (DCHFDA), propidium iodide (PI), resazurin, phorbol 12-myristate 13-acetate (PMA), Phalloidin Atto-633, accutase, fluorescent latex microspheres (2 μm), human AB serum, LPS from *E. coli* O111: B4, antibodies FITC anti-human CD11c, FITC anti-human CD14, FITC anti-human CD45, and other chemicals were purchased from Sigma-Aldrich (St. Louis, MO, USA).

### 2.2. Protocol of izTRAIL Preparation

To obtain a soluble trimeric form of the izTRAIL protein, the isoleucine zipper motif and the izTRAIL gene were synthesized and cloned into the pET101 plasmid vector (Novagen, Madison, WI, USA). The resulting gene was used to transform *E. coli* BL21 (DE3) cells, and a trimeric form of izTRAIL with a molecular weight of 80 kDa was obtained by microbial synthesis, followed by purification by metal-affinity chromatography [16].

### 2.3. Cell Cultures

Human acute myeloid leukemia cell line THP-1 was obtained from the ATCC (Manassas, VA, USA). Cells were cultured in RPMI 1640 medium supplemented with 10% fetal bovine serum (FBS), 40 μg/mL gentamicin sulfate, and 0.05 mM 2-mercaptoethanol at 37 °C in a humidified atmosphere of 5% CO_2_.

THP-1ad cells were derived from a subpopulation of THP-1 cells adhered to the surface of culture flasks. For this purpose, THP-1 cells were grown for 7–8 days after seeding at a concentration of 5 × 10^4^ cells/mL, until the appearance of cells attached to the surface of the flasks. Then, unattached cells were removed along with the medium, attached cells were washed with medium, and such cells were cultured for about 10 days with replacement of the growth medium with a fresh medium every 3 days until the forming of confluent cell culture of the new subline, THP-1ad. THP-1ad cells were cultured in RPMI/F12 and supplemented with 10% FBS and 40 μg/mL gentamicin sulfate, at 37 °C in a humidified atmosphere of 5% CO_2_. Accutase cocktail was used to detach THP-1ad cells from surface of culture flasks.

Macrophage-like THP-1PMA cells were obtained by treatment of THP-1 cells with PMA. THP-1 cells were cultured in RPMI/F12 supplemented with 10% FBS and 100 nM PMA for 96 h. Then, cells were washed three times with RPMI/F12 and used for experiments.

Peripheral blood-derived macrophages (PBDM) were derived from human monocytes. Monocytes were obtained from Cell Applications, Inc. (San Diego, CA, USA). The monocytes were cultured in DMEM supplemented with 10% FBS and 40 μg/mL gentamicin sulfate, at 37 °C in a humidified atmosphere of 5% CO_2_. The culture medium was replaced with a fresh medium 3 days after cell seeding and with DMEM supplemented with 2% FBS after the following 4 days. In 7 days of the monocyte cultivation in low FBS medium, they were used in experiments. Accutase cocktail was applied to detach macrophages from the surface of culture flasks.

Testing of cell cultures for mycoplasma infection was performed using the MycoFluor™ mycoplasma detection kit. Infection of cell cultures with mycoplasma was not detected.

### 2.4. Cell Proliferation and Cell Viability Assays

Cells were seeded in 96-well plates at a concentration of 5 × 10^3^ cells per well in 100 μL of growth medium and cultured in a CO_2_ incubator. The number of cells in suspension after their detachment with accutase and their viability were analyzed using a BD Accuri C6 flow cytometer (BD Bioscience, Franklin Lakes, NJ, USA). Cell viability was assessed after staining them in suspension in culture medium with 200 nM Calcein AM fluorescent dyes and 1 μg/mL propidium iodide [17]. The proliferative activity of cells was also assessed using DNA content analysis [18], and the expression of the Ki-67 nuclear antigen [19] was analyzed by the flow cytometer and the mitotic activity of cells. To estimate cellular DNA content, cells were suspended in phosphate-buffered saline, fixed with 70% ethanol, and stained with 1 μg/mL propidium iodide. To analyze the expression of Ki-67, we used PE anti-human Ki-67 antibodies. Control cells were stained with PE Mouse IgG1 k isotype Ctrl. Cell cycle was analyzed using ModFit LT 4.1 software [20] (Topsham, ME, USA). The mitotic cells were assessed by means of fluorescence of cells stained with nuclear dye H-33342 at a concentration of 1 μg/mL, and counting the number of mitotic cells using a DM 6000 fluorescence microscope (Leica, Wetzlar, Germany) [16,21]. The total number of analyzed cells in randomly selected fields was at least 500.

### 2.5. Short Tandem Repeat (STR) Profiling

STR loci and the amelogenin sex-determining marker were amplified using the COrDIS plus kit (Gordiz, Moscow, Russia) (detecting amelogenin, D5S818, D21S11, D7S820, CSF1PO, D2S1338, D3S1358, vWA, D8S1179, D16S539, TPOX, TH01, D19S433, D18S51, FGA, and D13S317) according to the manufacturer’s instructions in a GeneAmp^®^PCR system 9700 (Thermo Fisher Scientific, Waltham, MA, USA). Electrophoretic analysis was performed using a 3730/3130xl DNA Analyzer (Thermo Fisher Scientific, Waltham, MA, USA). After electrophoresis, the data were analyzed with Gene Mapper^®^ ID-X Software v1.5 [22] (Thermo Fisher Scientific, Waltham, MA, USA) to categorize peaks by size in relation to an internal standard allelic ladder.

### 2.6. Microarray-Based Comparative Genomic Hybridization (aCGH) Assay

DNA copy number profiling was performed using Agilent Sure Print G3 Human CGH microarray 180K (Agilent Technologies, Santa Clara, CA, USA). All experiments were conducted according to the manufacturer’s instructions. In brief, 1 µg of each genomic DNA from THP-1 parental cells and THP-1ad cells was labeled with Cy5-dCTP (Sigma-Aldrich, Hamburg, Germany) and reference human DNA was labeled with Cy3-dCTP (Sigma-Aldrich, Hamburg, Germany). Labeled DNA was applied to the array with hybridization buffer and human Cot-1 DNA (Thermo Scientific, Waltham, MA, USA). Array slides were incubated for 24 h at 65 °C. After washing and scanning the arrays, images were analyzed with Feature Extraction software v10.7.3.1 (Agilent Technologies, Santa Clara, CA, USA) [23]. Probe mapping was conducted according to its genomic location in the UCSC genome browser (human NCBI37/hg19). The Rank Segmentation statistical algorithm in NEXUS software v7.5 (Biodiscovery Inc., El segundo, CA, USA) was used to define copy number alterations in each sample.

### 2.7. Cell Viability Assay

To analyze TRAIL-cytotoxicity, cells obtained from growing cultures were seeded in 96-well flat-bottom culture plates at 5 × 10^3^ cells in 100 μL of growth medium per well. The recombinant protein izTRAIL was added 24 h after cell seeding into wells. The cytotoxicity was assessed by the ratio of the number of living cells in the experimental and control (not treated with izTRAIL) cultures 24 h after the addition of izTRAIL. The number of living cells was assessed by resazurin reduction after 48 h incubation with izTRAIL. Resazurin was added to the cells at a concentration of 30 μg/mL. Then cells were incubated with a dye for 4 h at 37 °C and 5% CO_2_, and the fluorescence intensity was measured at Ex.532 nm/Em.590 nm using an Infinite F200 plate reader (Tecan, Männedorf, Switzerland).

### 2.8. Cell Morphology Assay

Analysis of cell morphology was performed using an Eclipse Ti-E microscopic station (Nikon, Tokyo, Japan). To analyze the actin cytoskeleton, cells were fixed in 4% parapharmaldehyde solution, permeabilized for 10 min in intracellular staining permeabilization wash buffer (Biolegend, San Diego, CA, USA), and were stained with Phalloidin Atto-633 (1 nM), Hoechst 33342 (1 μg/mL), and Calcein AM (0.2 μM) for 25 min, in the dark, at room temperature. The analysis was performed using a TCS SP5 confocal laser scanning microscope (Leica, Wetzlar, Germany).

### 2.9. Cell Adhesion Assay

THP-1ad cells were seeded in the amount of 3 × 10^4^ per well of a 96-well plate in 100 μL of growth medium supplemented with anti-αVβ3 monoclonal antibody (ab78289, Abcam, Cambridge, UK) or anti-αVβ5 monoclonal antibody (MAB2528, R&DSystems Minneapolis, MN, USA), or with isotypic control to these antibodies (FITC anti-mouse IgG1, Biolegend, San Diego, CA, USA) at a concentration of 17 μg/mL, or with Cilengitide (Selleckchem, Pittsburgh, PA, USA) [24] at concentration of 20 μM. The cells were incubated for 1 day with the agents in a CO_2_ incubator. The analysis of cell adhesion was performed by counting nuclei stained with SYBER Green, using ImageXpress^®^ Micro XL (Molecular Devises, San Jose, CA, USA), before and after washing away non-adhered cells.

### 2.10. Cell Immunophenotyping

To study the expression of monocyte/macrophage markers (CD), cells were harvested from culture flasks, washed in cell staining buffer of 300 g for 5 min. Staining was performed using the following panel of monoclonal antibodies: APC anti-human CD11b, FITC anti-human CD11a, FITC anti-human CD11c, FITC anti-human CD14, FITC anti-human CD45, FITC Mouse anti-human CD163, FITC anti-human CD68, PE anti-human CD284, PE anti-human CD36, PE anti-human CD33, and PE anti-human HLA-DR. To determine nonspecific binding, cells were stained with the following isotype control antibodies: APC Mouse IgG1, k isotype Ctrl, FITC Mouse IgG1, k isotype Ctrl, PE Mouse IgG1 k isotype, and PE Mouse IgG2a k isotype. The staining was carried out at room temperature in the dark for 30 min. After staining, the cells were fixed with 2% paraformaldehyde solution. Analysis of CD expression was performed using a BD Accuri C6 flow cytometer. Histograms of expression of surface CD markers were formed using the FlowJo v10 software.

### 2.11. Phagocytosis Assay

The phagocytic activity was assessed after 2 h of cell incubation in growth medium supplemented with 1 mg/mL pHrodo Green *E. coli* [25]. To control the nonspecific staining, cells were incubated with 10 μg/mL of cytochalasin D for 30 min in a CO_2_ incubator, then 1 mg/mL of pHrodo Green *E. coli* was added, and the incubation was continued for another 2 h. Fluorescence was measured with a BD Accuri C6 flow cytometer. Phagocytosis was also studied using fluorescent latex microspheres (2 μm), opsonized in human AB serum for 30 min, and then incubated with cells. The analysis of the particle phagocytosis was carried out using a DM 6000 fluorescence microscope. To activate phagocytosis, cells were cultured in a growth medium supplemented with 10 μg/l of LPS from *E. coli* O111: B4 for 24 h. Phagocytic activity was characterized by percentage of fluorescent cells (phagocytic index) and by the average fluorescence intensity per cell (phagocytic number) in fluorescent cells [26].

### 2.12. Intracellular ROS Assay

Inducible and constitutive intracellular oxidative activity was assessed using a DCFH-DA probe (Ex 485 nm/Em 530nm) [27,28]. To evaluate the inducible oxidative activity, the cells were incubated in a growth medium with LPS from *E. coli* O111, as follows: B4 added at a concentration of 10 μg/mL for 24 h, washed with a fresh growth medium, and then incubated in the medium with DCFHDA (40 μM) for 15 min in a CO_2_ incubator. To assess the constitutive oxidative activity, the cells were incubated in a growth medium without LPS for 24 h, then washed with growth medium and incubated with 40 μM DCFHDA for 15 min. After staining, the cells were washed three times with PBS and cell fluorescence was analyzed using a BD Accuri C6 flow cytometer.

### 2.13. Intracellular NO Assay

To assess the intracellular NO activity, cells were stained with 5 μM DAF-FM DA [29] and incubated for 40 min in a CO_2_-incubator. Then, they were washed with a fresh growth medium and incubated for an additional 30 min in a CO_2_-incubator. To study the inducible NO production, cells were preincubated with 10 μg/mL LPS from *E. coli* O111: B4 for 24 h. Cell fluorescence was analyzed using a BD Accuri C6 flow cytometer.

### 2.14. Multiplex Analysis of Cytokine Production

The culture medium was taken away one day after cell seeding at the concentration of 5 × 10^4^ cells/mL, centrifuged (300× *g*, 5 min), and the supernatant was used for analysis. The content of cytokines in a supernatant was evaluated with a commercial Bio-Plex Pro Human Cytokine Grp I Panel 27-plex kit [30] (Bio-Rad, Hercules, CA, USA) using a Bio-Plex MAGPIX multiplex analyzer (Bio-Rad, Hercules, CA, USA).

### 2.15. Analysis of TRAIL Receptor Expression

To analyze the expression of TRAIL receptors, cells were harvested from culture flasks and washed in a cell staining buffer. Staining was performed using a panel of monoclonal antibodies, including APC anti-human CD261 (DR4), PE anti-human CD262 (DR5), Alexa Fluor 647 anti-human CD263 (DcR1), and PE anti-human CD264 (DcR2). To evaluate nonspecific binding, cells were stained with Human TruStain FcX (Fc receptor blocking solution) and isotype control antibodies APC Mouse IgG1 k isotype Ctrl, PE Mouse IgG1 k isotype Ctrl, and Alexa Fluor 647 Mouse IgG1 k isotype Ctrl. The staining was carried out at room temperature in the dark for 30 min. After staining, the cells were fixed with 2% paraformaldehyde solution. Expression analysis was performed using a BD Accuri C6 flow cytometer

### 2.16. Statistical Analysis

Results are presented as the mean ± standard deviation (M ± SD). Each experiment was carried out at least three times (*n* ≥ 3). The statistical significance of the results was determined by one-way ANOVA followed by multiple Holm–Sidak comparisons, *p* < 0.05.

## 3. Results

### 3.1. Derivation and Characteristics of THP-1ad Cells

**Figure 1:**
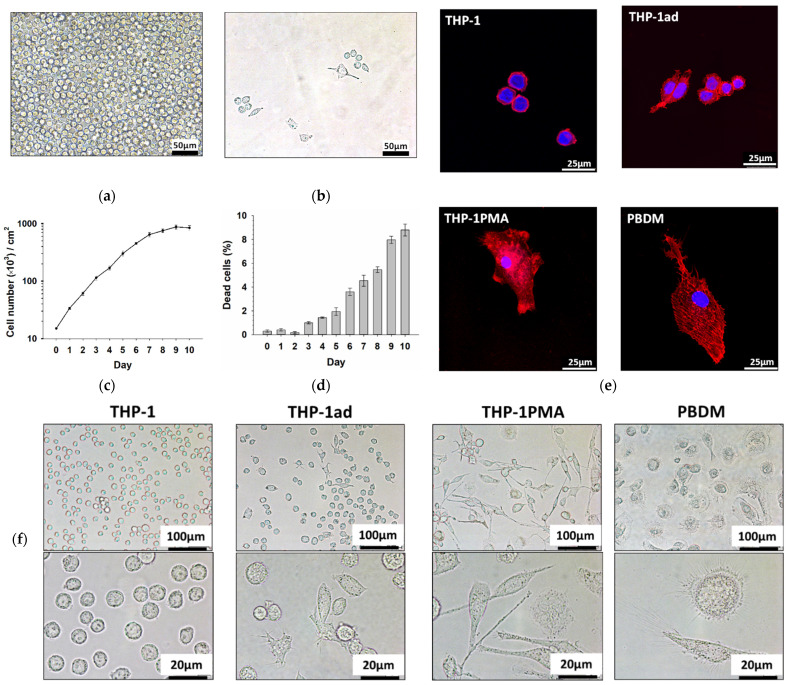
Micrographs of THP-1 cell cultures in day 7 of cultivation before and (**a**); After removal of non-attached cells (**b**); Growth curve of THP-1 cell culture (**c**); Increase in the percentage of nonviable THP-1 cells during the culture growth (**d**); Confocal microscopy. Micrographs of THP-1, THP-1ad, THP-1PMA, and PBDM cells. Staining with Hoechst 33342 (nuclear DNA, blue) and Phalloidin Atto 366 (actin, red) (**e**); Micrographs of THP-1, THP-1ad, THP-1PMA, and PBDM cells, 1 day after cell seeding at a density of 1.5 × 10^4^ cells/cm^2^ (**f**).

THP-1 AML cells were non-attached to the culture dishes at the initial stage of culture growth. A small number of adhered cells (not more than 1%) appeared only in seven days of cultivation when the density of a culture achieved about 6 × 10^5^ cells/cm^2^ (Figure 1a,b). It was from this time that a significant increase in the percentage of dead cells and the inhibition of the growth of the cell culture was revealed (Figure 1c,d), which indicates stressful conditions in the pericellular microenvironment. The non-adhered cells were removed by the washing of the culture with growth medium, and the adhered cells were cultured further. As a result of this procedure, a new cell line THP-1ad was derived, which is able to adhere to and grow on extracellular matrix. We received this cell line five times, and the results of all of the experiments are presented as the mean of three independent replicates.

In order to characterize the THP-1ad cells, we compared the morphological features of the THP-1ad and THP-1 cells, as well as that of the THP-1 cells differentiated to the macrophage-like phenotype by PMA (THP-1 PMA), and of the macrophages PBDM. The microscopic analysis showed that about 90% of the THP-1ad cells grew attached to the surface of culture dishes, forming islets of cells, which merged during cultivation and formed a confluent monolayer. A population of floating cells was also observed, however, its share was no more than 10%. The morphology of the THP-1ad cells was distinctly different from that of the THP-1 cells (Figure 1f). Some of the attached THP-1ad cells had a round shape, and some were spread. Some of the spread THP-1ad cells had a polarized phenotype and “spindle shape”, while the other cells had filopodia and pseudopodia, but did not have a polarized shape (Figure 1f). The THP-1 PMA and PBDM cells were larger than the THP-1ad cells, and their “spindle shape” phenotype was more pronounced than that in THP-1ad cultures (Figure 1f). The morphological features of the THP-1ad cell culture were stable during long-term subcultivation with cell seeding at a density of 1.5 × 10^4^ cells/cm^2^ and growth for three days.

The confocal microscopy analysis of cells stained with phalloidin and H-33342 showed a difference in the structure of the actin cytoskeleton in the THP-1ad cells in comparison with THP-1 (Figure 1e). The THP-1 cells contain actin mainly in the cortical layer of the cytoplasm near the plasma membrane (Figure 1e). In the THP-1ad cells, as well as in THP-1PMA, and PBDM, the actin cytoskeleton is located throughout the cytoplasm, but not uniformly. At the same time, it is clearly seen that the distribution of actin in the cytoplasm of the THP-1ad and THP-1PMA cells is more diffuse and less structured than in PBDMs, which contained parallel-oriented strands of actin microfilaments (Figure 1e). The results obtained indicate the initiation of the program of actin reorganization accompanied by the forming cytoskeleton in the THP-1ad and THP-1PMA cells, however, this program non-adequately simulates actin cytoskeleton of PBMD.

The genetic study of the THP-1ad cells, in comparison with THP-1 cell line, was performed using the short tandem repeat (STR) analysis, which allows the evaluation of the repetition of alleles of certain loci in DNA. The analysis of the identified genotypes was carried out in comparison with the ATCC reference base. It was found that the genetic profile of the THP-1ad cells matched by 93% with the profile of the parental acute monocytic leukemia cell line THP-1 from ATCC (Table 1). The THP-1ad and THP-1 cells differ only by two microsatellite loci, diallelic AMEL locus and D13S317 locus. It was also confirmed that the genetic profile of the THP-1 cells we used was completely identical to that of THP-1 cells from the ATCC base (Supplement, Table S1). The results of STR genotyping THP-1ad cells using an expanded set of DNA loci are presented in Supplement (Table S2, Figure S1).

In addition to the DNA changes detected in the THP-1ad cells in comparison with the parental THP-1 cell line by the STR method, specific chromosomal aberrations were also revealed using the aCGH assay (Table S3). It was found that, in contrast to THP-1 cells, the THP-1ad cells have deletions in 3 and X chromosomes, monosomy on the Y chromosome, and duplications in 1, 5, 6, 7, 8, 10, 11, 17, 18, and 20 chromosomes. Heterozygosity in the THP-1ad cells is present on chromosomes 9 and 10.

Thus, the leukemic THP-1ad cells are related to THP-1 cells. They derived from the THP-1 cells and have chromosomal aberrations different from those of the parental cell line. The possibility of the contamination of the THP-1 cell line used with other cell lines is excluded completely.

### 3.2. Adhesion of THP-1ad Cells to Extracellular Matrix Is Mediated by Integrin aVβ5

**Figure 2:**
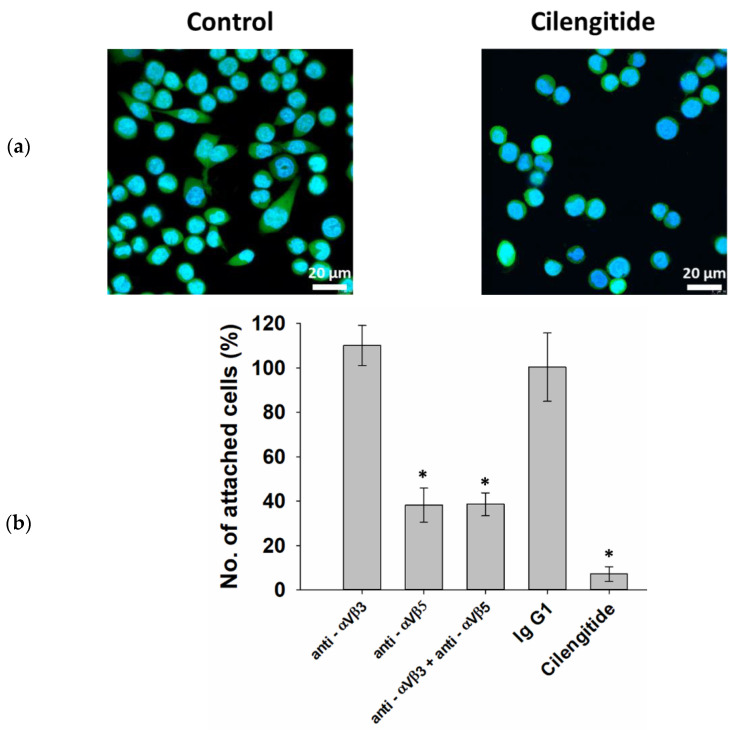
Micrographs of THP-1ad cells after 1 day of incubation with 20 μM of Cilengitide and without Cilengitide (control); staining of the cytoplasm with Calcein AM (green), and of cell nuclei with Hoechst 33342 (blue) (**a**). Percentage of THP-1ad cells attached to the surface of culture dishes 1 day after incubation with anti-αVβ3 and anti-αVβ5 antibodies, with isotypic control Ig G1, and Cilengitide relative to control (90% of attached cells) (**b**). *—*p* < 0.05 in comparison with control.

It is known that serum proteins can adhere to a surface of culture dishes, resulting in the formation of an extracellular matrix (ECM) [31]. An ECM contains adhesive proteins including fibronectin [32]. Cell binding to the main adhesive protein of the ECM, fibronectin, is mediated by integrins of the αV group and, to a lesser extent, of α5β1 [33]. In order to assess the contribution of the αV group integrins to the adhesion of the THP-1ad cells to the extracellular matrix, we studied the effect of Cilengitide on the adhesion of the THP-1ad cells in the culture dishes. Cilengitide is the RGD peptide that blocks the binding of αV, a subunit of cell membrane integrins, to the ECM proteins [34]. It was found that the incubation of the THP-1ad cells, together with 20 µM of Cilengitide for 24 h, leads to morphological changes, and the cells acquire a round shape and detach from the surface of the dishes (Figure 2). We tried to elucidate quantitatively the role of integrins αVβ3 and αVβ5 in the adhesion of the THP-1ad cells to the extracellular matrix. For this, the cells were incubated for 24 h with the anti-αVβ3 monoclonal antibody, the anti-αVβ5 monoclonal antibody, a combination of anti-αVβ3 and anti-αVβ5, as well as with isotypic control to these antibodies (FITC anti-mouse IgG1) and Cilengitide (Figure 2b).

The blocking of αVβ3 integrin with antibodies did not inhibit the adhesion of the THP-1ad cells to the ECM. The blocking of integrin αVβ5, as well as the combined blocking of αVβ3 and αVβ5, led to a decrease in cell adhesion by more than 2.6 times (Figure 2b). Cilengitide almost completely inhibited the cell attachment to the ECM. These results indicate that integrin αVβ5, but not αVβ3, is involved in the adhesion of the THP-1ad cells to the ECM. However, since blocking αVβ5 does not lead to the detachment of all of the cells from the matrix, one can assume not only αVβ5, but also some other integrins are also involved in the adhesion of the THP-1ad cells to the ECM.

### 3.3. THP-1ad Cells Have a Similar Immunophenotype to Macrophages

The immunophenotyping of the THP-1ad cells was performed for 12 major markers of monocyte/macrophage differentiation in comparison with THP-1, THP-1PMA, and PBDM cells. In addition to acquiring macrophage-like morphology, the THP-1ad cells also contain immunophenotypic markers pointed to the formation of a macrophage-like phenotype. The immunophenotype of the THP-1ad cells in comparison with other cell types is presented in Table 2. The THP-1ad cells are positive for the main macrophage markers CD11b +/CD11c +/CD14 +/CD284 +, which are absent in the parental THP-1 cells (Table 2 and Figure S2). The percentage of the population expressing CD14 and CD284 in the THP-1ad cells does not differ from that of the PBDM macrophages. In addition, the percentage of the THP-1ad cells expressing HLA-DR increased to 60 ± 2%.

### 3.4. THP-1ad Cells Are Capable of High Phagocytic Activity of pHrodo Green E. coli

**Figure 3:**
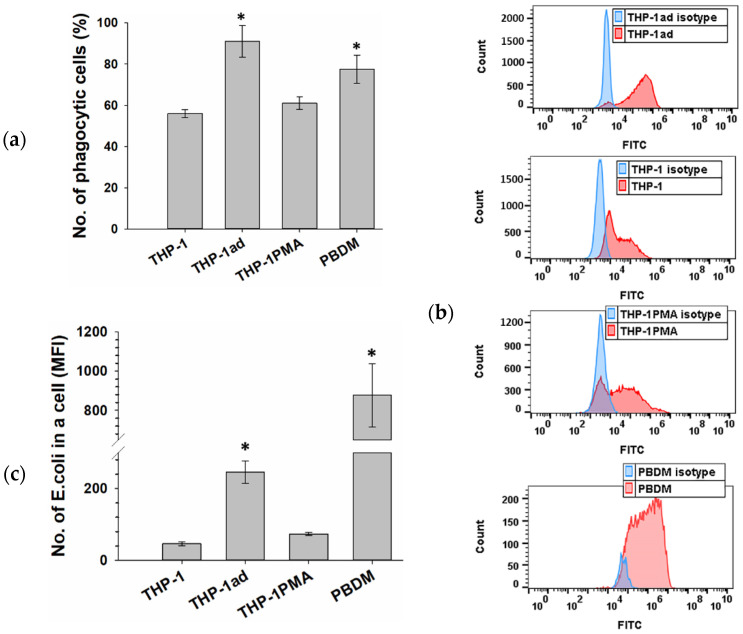
Phagocytic index (% of phagocytic cells) to pHrodo Green *E. coli* (**a**); Histograms of phagocytic activity (control isotype—blue, cells incubated with pHrodo Green *E. coli*—red) and (**b**); Phagocytic number (number of phagocytosed pHrodo Green *E. coli* per a cell, expressed in arbitrary units of MFI, mean fluorescence intensity) (**c**); *—*p* < 0.05 in comparison with THP-1.

The presence of membrane receptors CD14 and CD284 in the THP-1ad cells, which are part of the multimolecular receptor complex that recognizes the lipopolysaccharide of the bacteria cell wall, suggests the ability of the THP-1ad cells to perform phagocytosis. The THP-1ad cells, similar to the parent THP-1 cells, were unable to phagocyte latex microspheres both constitutively and after stimulation by LPS (Figure S3), in contrast to the PBDM and THP-1PMA cells. At the same time, the THP-1ad cells phagocytosed the pHrodo Green *E. coli*. Most of the THP-1ad cells (90 ± 6%) possessed phagocytic activity against the *E. coli* preparation, which was higher (*p* < 0.05) than that of the THP-1 and THP-1PMA cells (Figure 3a).

It should also be noted that the phagocytic number of the THP-1ad cells, determined by the number of phagocytosed particles (fluorescence intensity), was also higher (*p* < 0.05) than those of the THP-1 and THP-1PMA cells (Figure 3b,c). Thus, the THP-1ad cells are characterized by high phagocytic activity of *E. coli* bacteria, similar to macrophages PBDM, but not to the latex microparticles, in contrast to PBDMs.

### 3.5. ROS and NO Production Is Activated in THP-1ad Cells as Well as Macrophage Cells

Another metabolic characteristic that can change during the acquisition of a macro-phage-like phenotype by the THP-1ad cells is the production of reactive oxygen species (ROS), which is one of the main properties of phagocytes. Macrophages activated by LPS are characterized also by increased production of nitric oxide (NO). The analysis of constitutive and LPS-inducible oxidative activity and NO production in cells was carried out according to the fluorescence intensity of DCFHDA [35] and DAF-FM DA [27] probes, respectively.

**Figure 4:**
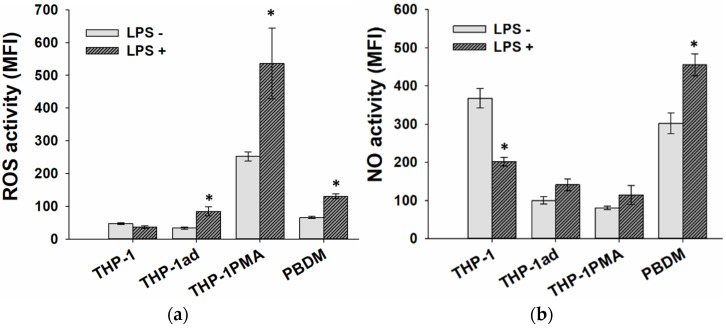
Constitutive and LPS-inducible oxidative activity (**a**) nitric oxide activity (**b**) in THP-1ad cells in comparison with THP-1 cells, THP-1PMA, and PBDM cells. MFI is the mean fluorescence intensity of cells (arb.units) loaded with DCFHDA and (**a**) DAF-FM DA (**b**). *—*p* < 0.05 in comparison with corresponding LPS- cells.

The common features of the THP-1ad and THP-1PMA cells, as well as for macrophages PBDM, was an increase in the intracellular ROS activity and the NO production in replay to adding LPS, in contrast to the THP-1 cells (Figure 4). This feature also points to acquiring macrophage-like signs by the THP-1ad cells.

### 3.6. Cytokine Secretion by THP-1ad Cells Is Similar to Macrophages

**Figure 5:**
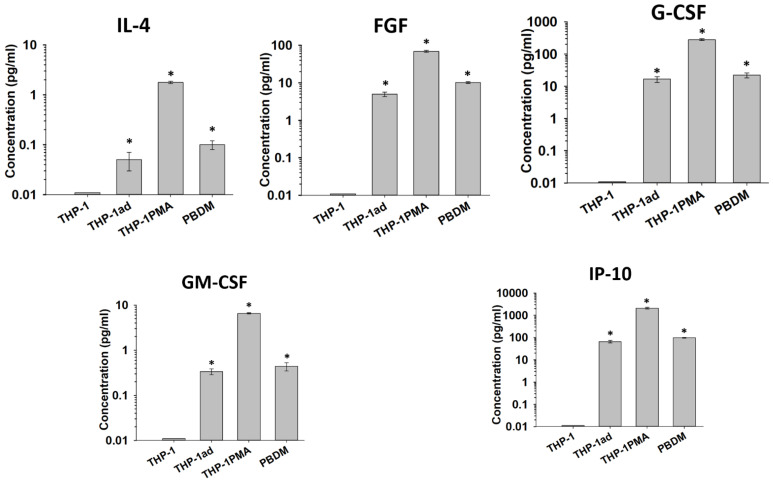
Analyses of cytokine production by THP-1, THP-1ad, THP-1PMA cells, and PBDM. Y axis: Concentration of the cytokines in a culture media a day after cell seeding at 5 × 10^4^ cells/cm^2^. *—*p* < 0.05 in comparison with THP-1.

The analysis of the cytokine profile of the THP-1ad cells in comparison with the parental THP-1, THP-1PMA cells, and PBDM can characterize the differentiation status of the studied cells. We performed a multiplex analysis of the constitutive production of 27 cytokines by the THP-1ad cells in comparison with THP-1, THP-1PMA, and PBDM (Table S3). In contrast to the parental THP-1 cells, the THP-1ad cells were found to be characterized by the production of IL-4, FGF, G-CSF, GM-CFF, IP-10, and MIP-1α, which is also characteristic of the THP-1PMA cells and PBDM. Moreover, the level of IL-4, FGF, G-CSF, GM-CSF, and IP-10 in the THP-1ad cell cultures was closed to that of PBDM, but not of the THP-1PMA cells (Figure 5). The constitutive production of eight cytokines by the THP-1ad cells, including IL-2, IL-5, IL-6, IL-17a, MCP-1, MIP-1β, TNF-α, and VEGF, differed significantly (*p* < 0.05) from that of the parent THP-1 cells and increased to the level characteristic of PBDMs (Figure S4). The THP-1PMA cells produced the most intensive of all of the cytokines relative to the THP-1, THP-1ad cells and PBDM.

Thus, the cytokine production point to the macrophage-like phenotype formation of the THP-1ad cells.

### 3.7. The Proliferative Activity of Macrophage-like THP-1ad Cells Does Not Differ from Parental Cells

It is known that cell treatment with an exogenous inducer of cell differentiation suppresses the cellular proliferation [36]. According to our results acquiring the signs of a macrophage-like phenotype, the THP-1ad cells did not lose the ability of proliferation.

**Figure 6:**
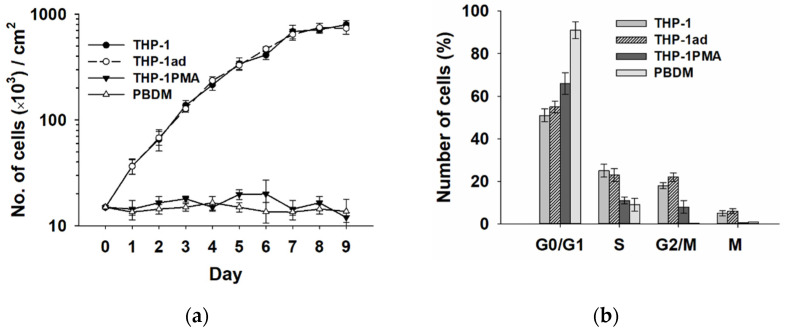
Growth curve of THP-1ad cell culture and (**a**) distribution of THP-1ad cells on cell cycle phases one day after their seeding (**b**) in comparison with THP-1, THP-1PMA and PBDM cells.

The analysis of culture growth by cell counting (Figure 6a) indicated that duplication time of cell number in the first three days of the THP-1ad cell cultivation was 24 ± 2 h, as for the THP-1 cells (24 ± 1 h). The distributions of growing the THP-1ad and THP-1 cells in the cell cycle were identical. The proportion of mitotic cells in the THP-1ad cultures a day after seeding was 6.0 ± 1.1% did not differ significantly from the indicator of the THP-1 cells (6.7± 0.8%). As expected, the cell proliferation and the mitotic cells in the THP-1PMA and PBDM cell cultures were not revealed, and these cells accumulated predominantly in the G1 phase of the cell cycle. The expression of Ki-67 antigen, another marker of cell proliferation activity, was revealed in 98 ± 2% and 99 ± 1% of the THP-1ad and THP-1 cells, respectively, and only in 29 ± 7% of the THP-1PMA cells. Ki-67-positive PBDMs were not found.

Thus, the THP-1ad cells, acquiring a macrophage-like phenotype retain high proliferative activity in contrast to the THP-1PMA cells.

### 3.8. Macrophage-like THP-1ad Cells Acquire Resistance to TRAIL Induced Cell Death

**Figure 7:**
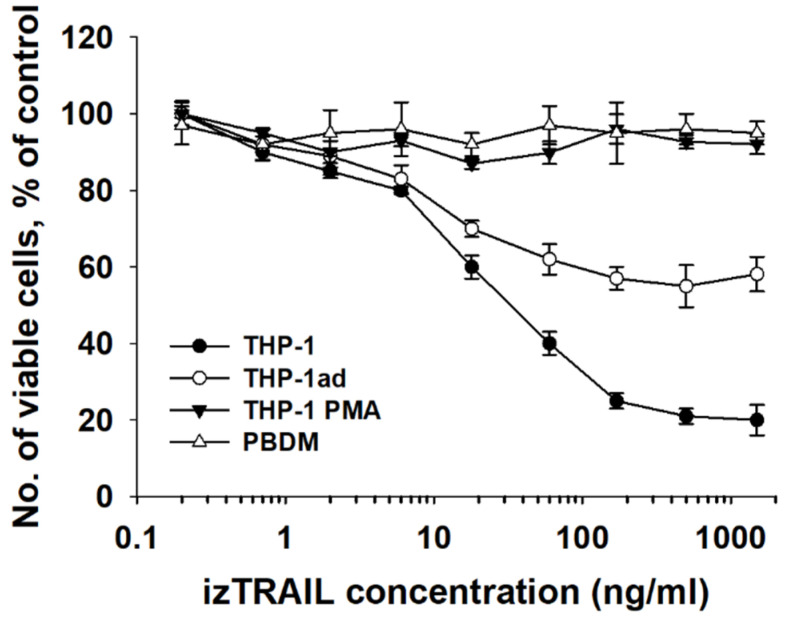
Cytotoxic effects of izTRAIL on THP-1ad, THP-1, THP-1PMA cells, and PBDM. The ordinate is the number of living cells in percentage relative to the control (non-treated cultures) a day after the addition of izTRAIL.

It is known that the differentiation of leukemic cells by exogenous factors can initiate their resistance to TRAIL-mediated apoptosis [8,9,10,11]. We assumed that the appearance of a macrophage-like phenotype in the THP-1ad cells might be accompanied by an increase in their resistance to TRAIL-induced cell death. In this study, the effect of izTRAIL on the THP-1ad, THP-1, THP-1PMA cells, and PBDM was compared (Figure 7).

It was shown previously that the izTRAIL protein induced death in the THP-1 cells [16]. The protein izTRAIL was found to have no toxic effect, even at high concentrations (up to 1.5 μg/mL), on the macrophage-like cells THP-1PMA and PBDM, indicating their TRAIL-resistance. The data in Figure 7 show two subpopulations in each of the cell cultures, THP-1ad and THP-1.

One of the subpopulations was sensitive to the cytotoxic effect of izTRAIL, and the other was TRAIL-resistant, since a significant portion of living cells was detected at high concentrations of izTRAIL (Figure 7). The percentage of TRAIL-insensitive cells was 20 ± 5% and 60 ± 5% in the THP-1 and THP-1ad populations, respectively. The IC50 value of the izTRAIL protein (the concentration of the agent at which the number of cells in TRAIL treated cultures was two times less than in the non-treated cultures) for the TRAIL-sensitive subpopulation of the THP-1 cells (80 ± 5%) was of 20 ± 7 ng/mL. The IC50 value of the izTRAIL protein for the TRAIL-sensitive subpopulation of the THP-1ad cells (40 ± 5%) was of 10 ± 4 ng/mL (Figure 7). The difference of these IC50 values was insignificant.

Thus, the results obtained show a decrease in the TRAIL-sensitivity of the THP-1ad cell population as compared to the THP-1 cells due to an increase in the percentage of the subpopulation that was resistant to TRAIL-induced cell death.

### 3.9. Resistance of THP-1ad Cells to TRAIL Is Associated with a Decrease in Expression of Death Receptors DR4 and DR5

**Figure 8:**
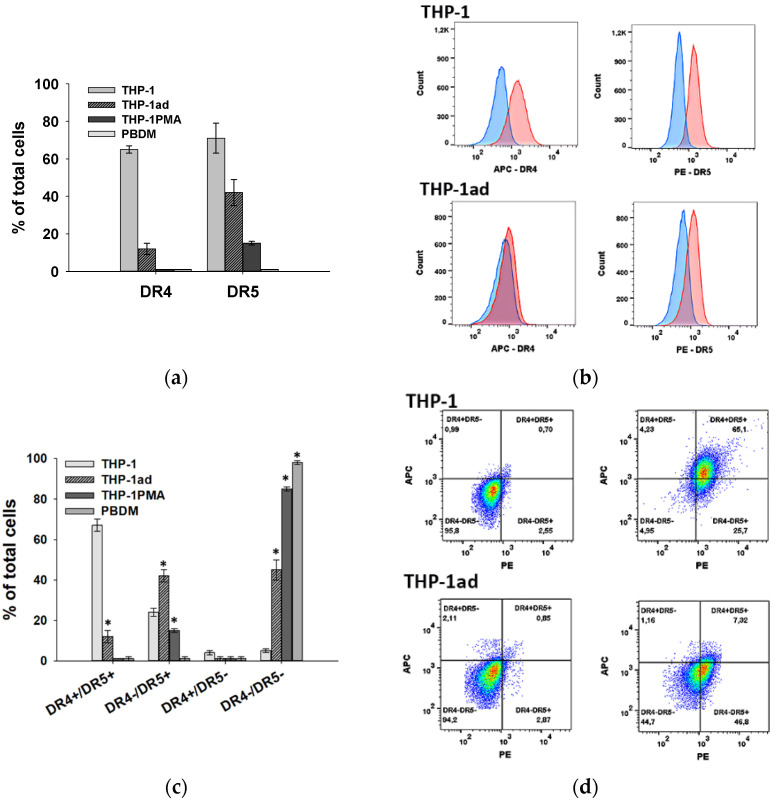
Percentage of cells expressing DR4 and DR5 in populations of THP-1, THP-1ad, THP-1PMA cells, and PBDM (**a**); *—*p* < 0.05 in comparison with THP-1. Histograms of cells stained with APC Mouse IgG1 k isotype Ctrl or PE Mouse IgG1 k isotype Ctrl (control isotypes—blue), or with monoclonal antibodies APC anti-human CD261 (to DR4), or PE anti-human CD262 (DR5) (red) from flow cytometer (**b**); Percentage of cells in THP-1, THP-1ad, THP-1PMA, PBDM populations, which express both DR4 and DR5 (DR4+/DR5+), only DR4 (DR4+/DR5−), or only DR5 (DR5−/DR5+), and neither DR4 nor DR5 (DR5−/DR5−) (**c**); Scattergrams of THP-1 and THP-1ad cells stained with control isotypes (left), or with both monoclonal antibodies APC anti-human CD261 (DR4, FL2-H) and PE anti-human CD262 (DR5, FL4-H) (right) (**d**); *—*p* < 0.05 in comparison with THP-1.

We analyzed the expression of the TRAIL receptors in order to evaluate the mechanism of the increase in TRAIL-resistance of the THP-1ad cells compared to the THP-1 cells. The expression of antiapoptotic TRAIL receptors (DcR1 and DcR2) was not found in the studied cell types. The highest percentage of cells carrying the proapoptotic receptors DR4 and DR5, 65 ± 2% and 71 ± 8%, respectively, was revealed in the THP-1 cell population (Figure 8a). The significant decrease in the portion of THP-1ad cells expressing the receptors DR4 and DR5 of 12 ± 3% and 42 ± 7%, respectively, in comparison with the THP-1 cells was revealed. The complete loss of DR4+ cells and a decrease in the number of cells carrying DR5 to 15 ± 3% were detected afterwards in the macrophage-like differentiation of the THP-1 cells using PMA. No expression of both receptors DR4 and DR5 was found on the macrophages obtained from the peripheral blood monocytes (Figure 8a). The data on the expression of the TRAIL receptors are consistent with the cytotoxic effect of izTRAIL on the studied cells, which showed that 80% of the THP-1 cells and 40% of the THP-1ad cells are sensitive to izTRAIL, while all of the cells of PBDM and THP- 1PMA were totally resistant to TRAIL-induced cell death (Figure 8a).

We also analyzed the percentage of the subpopulations carrying both DR4 and DR5 (DR4+/DR5+), only DR4 (DR4+/DR5−), or only DR5 (DR4−/DR5+), as well as carrying none of these receptors (DR4−/DR5−) in the THP-1ad, THP-1, THP-1PMA cells, and PBDM populations (Figure 8c). For this purpose, the cells were stained with both antibodies to DR4 and DR5, and the shift of this population relative to the cells stained by two control isotypes was analyzed. It was found that there was a significantly (*p* < 0.05) lower percentage of DR4+/DR5+ cells and an increased percentage of DR5−/DR5+ cells in the THP-1ad population relative to the THP-1 cells. In addition, about 45% of the THP-1ad cells were DR4−/DR5−, in contrast to 5% of DR4−/DR5− cells in the THP-1 population (Figure 8c,d). At the same time, about 85% of the THP-1PMA cells and all of the PBDM macrophages were DR5−/DR5− (Figure 8c).

We have shown that resistance of the THP-1ad cells to TRAIL-induced cell death is associated with a decrease in the number of DR4- and DR5-positive cells. However, resistance to the cytotoxic effect of TRAIL may also be associated with a decrease in the expression of DR4 and DR5 receptors on the cell surface in populations of DR4+ and DR5+ cells. We evaluated quantitatively the expression of DR4 and DR5 receptors on the THP-1ad cells by means of the fluorescence intensity (MFI) per cell in two subpopulations (DR4+/DR5+ and DR5−/DR5+) in comparison with those on the THP-1 and THP-1PMA cells. For this, the total cell population stained with antibodies to DR4 and to DR5 was gated relative to the isotypic controls of these antibodies (Figure 8c). The MFI for the DR4+/DR5+ population was analyzed in the THP-1ad cells only in comparison with the THP-1, since this population is not represented in THP-1PMA. The MFI for the DR5−/DR5+ subpopulation of the THP-1ad cells was analyzed in comparison with both THP-1 and THP-1PMA. It turned out that the expression of DR4 and DR5 receptors per cell in the DR4+/DR5+ subpopulations on the THP-1ad and THP-1 cells was the same and the expression of DR5 receptors per cell in the DR5−/DR5+ subpopulations of the THP-1ad, THP-1PMA, and THP-1 cells (Figure S5). Since the DR4+/DR5− subpopulation is absent in the THP-1ad and THP-1PMA cells, and it is negligible in the THP-1 cells (<5%, Figure 8c), it can be concluded that the expression of the DR4 and DR5 receptors in all of the subpopulations of the THP-1ad, THP-1PMA, and THP-1 cells carrying these receptors are the same.

Thus, the increased TRAIL-resistance of the THP-1ad cells in comparison with THP-1 was consistent with a decrease in the subpopulations of cells carrying the DR4 and DR5 receptors, while the number of DR4 and DR5 on the cells bearing these receptors was not changed.

## 4. Discussion

The acquisition by leukemic myeloid cells of macrophage-like phenotype signs can increase their resistance to TRAIL-induced apoptosis [8,9,10,11]. Usually, this phenomenon for leukemic cells is described as a response to the action of exogenous differentiating factors.

Our work also showed that PMA, a well-known inducer of macrophage-like differentiation of THP-1 cells, significantly enhanced their resistance to TRAIL-induced cell death. However, in this work, we found the appearance of signs of a macrophage-like phenotype and an increase in TRAIL-resistance in the THP-1 acute myeloid leukemia cells, which was provoked in vitro in a high-density cell culture. The appearance of a macrophage-like phenotype in some of the cells of the THP-1 population was detected only during growth inhibition and increased in cell death after seven days of cultivation, when culture density achieved a high value of about 6 × 10^5^ cells/cm^2^. Apparently, this event was because seven days after cell seeding and subsequent growth of cell population, stress conditions were formed in the culture. This was indicated by a two-fold increase in the percentage of dead cells from six to seven days. By this time, acidification of the culture medium was observed, and the density of cell culture was so high that, due to diffusion limitations of mass transfer, one can expect stress in the cell microenvironment, in particular low pericellular pH 6.7–6.5 [37] and pericellular hypoxia [38]. The microenvironment stress-induced cell change was confirmed by the results of comparative genetic studies, such as STR and aCGH assay, in the THP-1ad and THP-1 cells. The population of THP-1 cells appeared to increase in their resistance to TRAIL-induced cell death together with the acquisition of a macrophage-like phenotype. The probability of the appearance of mutated cells is not high, which is confirmed by a small percentage of cells adhered to extracellular matrix in the culture (no more than 1%). However, the mutated cells attach to the extracellular matrix and maintain the same rate of proliferation as the parent cells. These two circumstances made it possible to derive the THP-1ad cell line by removing the suspension of unattached THP-1 cells and the subsequent growth of the remaining attached THP-1ad cells. Moreover, the combination of the increase in TRAIL-resistance and the retaining of a high proliferative ability of mutated myeloid leukemia cells in a high-density culture suggest a new dangerous mechanism for the tumor progression in vivo.

It should be noted that the signs of a macrophage-like phenotype in the THP-1ad and THP-1PMA cells are insufficiently adequate to those in the macrophages. This applies to the appearance of the morphological characters that do not fully correspond to those in macrophages, as well as to immunophenotypic signs, LPS-induced production of ROS and nitric oxide, and cytokine production. Perhaps the insufficiently complete manifestation of the signs of macrophage-like differentiation indicates the ability to retain proliferative potential in THP-1ad cells. In other words, since mutagenesis in acute myeloid leukemia cells is able to induce only a limited appearance of some signs, and not a full-fledged program of macrophage-like differentiation, proliferation can be retained in mutated macrophage-like cells, in contrast to macrophages.

In our results, attention is attracted the complete suppression of TRAIL-sensitivity in the non-proliferating THP-1PAM cells and retaining partially the TRAIL-sensitivity and full proliferative potential in the THP-1ad cell population. It is likely that, the endogenous mutagenic stimulus that caused the appearance of macrophage-like THP-1ad cells in the culture of the parent THP-1 cells is insufficient to activate the signaling pathways that suppress cell proliferation, in contrast to the THP-1PMA cells. Differentiation of the THP-1 cells by PMA is known to be accompanied by PKC-dependent induction of p21WAF1/Cip1 and stopping the cell cycle [36,39].

The results presented in this work indicate the heterogeneity of the THP-1 and THP-1ad cell populations in terms of their sensitivity to the cytotoxic action of izTRAIL. For example, about 20% of the cells in the THP-1 population were TRAIL-resistant, remaining alive even at the high concentrations of the protein izTRAIL, and 80% of the THP-1 cells were sensitive to the apoptogenic effect of izTRAIL, with IC50 about 20 ng/mL. An increase in the resistance to TRAIL-induced apoptosis in the cells of the THP-1ad subline is associated with an increase in the percentage of TRAIL-resistant cells three times, up to 60%. It is important to note that the inhibitory concentration IC50 of the protein izTRAIL for TRAIL-sensitive subpopulations of the THP-1 and THP-1ad cells did not differ significantly.

The presented results suggest that the decrease in sensitivity to the cytotoxic effect of TRAIL in the studied cells is determined by a decrease in the subpopulations bearing DR4 and DR5 receptors. The macrophages and the THP-1PMA cells are totally resistant to TRAIL-induced cell death and do not contain the subpopulations DR4+/DR5+ or DR4+/DR5−, although the THP-1PMA cells contain about 15% of DR5−/DR5+. The percentage of DR4-bearing THP-1ad cells was about 10% and included only subpopulations DR4+/DR5+. The share of DR5+ THP-1ad cells was 45%, including 10% of DR4+/DR5+, and 35% of DR5−/DR5+ cells. Therefore, the total share of the THP-1ad cells bearing proapoptotic TRAIL receptors DR4 and DR5 was of 45% (Figure 8) and was consistent to the percentage of the TRAIL-sensitive cells (Figure 7). The data on resistance to TRAIL, expression of TRAIL receptors, and the macrophage-like phenotype in the studied cells seem to indicate a relationship between the appearance of the signs of macrophage-like cells and the expression of TRAIL receptors. It should be noted that a difference between the loss of DR4 and DR5 receptors on the THP-1ad cells was observed. For example, the subpopulation DR5−/DR5+ increased from 25% to 35% compared to the THP-1 cells, and DR4+/DR5− disappeared totally in the THP-1ad cell population.

In order to understand the mechanism of the decrease in the sensitivity of cells studied to the cytotoxic effect of izTRAIL, it is important that quantitative expressions of DR4 and DR5 receptors on the THP-1 and THP-1ad cells bearing them were the same. Apparently, leukemic cells with macrophage-like differentiation either completely lose TRAIL receptors and become TRAIL-resistant or retain the expression of receptors providing TRAIL-induced cell death. The results obtained in this work on the key role of TRAIL receptors in the sensitivity of acute myeloid leukemia cells to TRAIL-induced cell death are consistent with the literature data. For example, it is known that in leukemic cells, TRAIL-induced cell death is triggered through DR4 [40,41]. It is also known that, unlike DR4, which is involved only in the initiation of apoptosis, the DR5 receptor can participate in the activation of survival, proliferation, and migration of tumor cells by action on the intracellular signaling pathways involving NF-κ B, PI3K/Akt, MAPK, and JNK [42]. Our results point to key role of DR4 and DR5 in reducing the sensitivity of human leukemic cells THP-1ad to TRAIL-induced cell death.

Taken together, our results suggest that the decrease in the cytotoxic effect of TRAIL on the THP-1ad cells in comparison with the THP-1 cells is determined by a decrease in the subpopulation of cells carrying DR4 and DR5 receptors without decrease in the expression of DR4 and DR5 on cells retaining these receptors.

## 5. Conclusions

Cells resistant to TRAIL-induced death may appear in high-density cultures of THP-1 AML cells. Such TRAIL-resistant THP-1ad cells have macrophage-like properties. The resistance of the macrophage-like THP-1ad cells to TRAIL-induced cell death is associated with a decrease in the expression of in receptors, DR4 and DR5. Thus, the macrophage-like phenotype formation with the maintenance of a high proliferative potential of leukemic cells caused by stress conditions in high-density cell cultures in vitro can induce an increase in resistance to TRAIL-induced cell death due to the loss of proapoptotic TRAIL receptors.

## Figures and Tables

**Table 1:** STR profile of THP-1ad cells compared to the reference THP-1 profile from ATCC base.

Loci	Genotype of THP-1ad Cells	Closest Match (THP-1 ATCC)
D5S818	11,12	11,12
D13S317	13,14	13,13
D7S820	10,10	10,10
D16S539	11,12	11,12
vWA	16,16	16,16
TH01	8,9,3	8,9,3
AMEL	X,X	X,Y
TPOX	8,11	8,11
CSF1PO	11,13	11,13

**Table 2:** Expression of CD markers of macrophage differentiation on THP-1ad cells in comparison with THP-1, THP-1 PMA and PBDM cells.

CD (Clusters of Differentiation)	THP-1	THP-1ad	THP-1 PMA	Macrophages (PBDM)
Integrin αL, (*CD11a*)	85 ± 9%	86 ± 1%	91 ± 2%	98 ± 2%
Integrin αM, (*CD11b*)	–	18 ± 2%	–	90 ± 3%
Integrin αX, (*CD11c*)	–	56 ± 3%	90 ± 1%	66 ± 1%
Co-receptor for LPS (*CD14*)	–	19 ± 1%	95 ± 1%	47 ± 2%
Siglec-3 (*CD33*)	99 ± 1%	99 ± 1%	99 ± 1%	99 ± 1%
SCARB3 (*CD36*)	–	–	20 ± 1%	97 ± 1%
PTPRC (*CD45*)	96 ± 8%	96 ± 1%	99 ± 1%	99 ± 1%
Fc-γ receptor 1 (*CD64*)	95 ± 1%	95 ± 1%	25 ± 3%	55 ± 4%
Macrosialin (*CD68*)	51 ± 3%	47 ± 5%	–	95 ± 4%
Receptor for the hemoglobin–haptoglobin complex (*CD163*)	–	–	–	–
TLR4 (CD284)	–	49 ± 13%	–	9 ± 1%
MHC II (*HLA-DR*)	21 ± 1%	60 ± 1%	–	59 ± 2%

## Data Availability

The data presented in this study are contained within this article and online supplement data.

## Supplementary Materials

The following supporting information can be downloaded at: https://www.mdpi.com/article/10.3390/biom12020150/s1, Table S1. STR profile of THP-1 cells in comparison with the reference profile of THP-1 from ATCC; Table S2. Comparison of STR profiles of THP-1ad and THP-1 cells for an expanded set of DNA loci compared to ATCC; Table S3. Specific chromosomal aberrations characteristic of THP-1ad cells, identified by the aCGH method; Table S4. Analyses of cytokine production by THP-1, THP-1ad, THP-1PMA, and PBDM cells; Figure S1. Electrophoregrams of STR-profiles of THP-1ad cells (a) and parent THP-1 cells (b) respectively; Figure S2. Histograms of expression of the main macrophage markers CD11b, CD11c, CD14, and CD284 by THP-1ad cells in comparison with THP-1 cells, THP-1PMA cells, and macrophages (PBDM). Isotype control is blue, antibodies are red; Figure S3. Inducible and constitutive phagocytic index (a) and phagocytic number of latex microspheres (b) of THP-1ad cells in comparison with THP-1 cells, THP-1 cells treated with PMA and PBDM; Figure S4. Analyses of cytokine production by THP-1, THP-1ad, THP-1PMA, and PBDM cells. Y axis: concentration of the cytokines in a culture media a day after cell seeding at 5 × 10^4^ cells/cm^2^. *—*p* < 0.05 compared to THP-1; Figure S5. Mean number of receptors DR4 and DR5 per cell in a subpopulation DR4+/DR5+ of THP-1ad cells in comparison with THP-1 cells (a); and mean number of receptors DR5 per cell in subpopulation DR5−/DR5+ of THP-1ad cells in comparison with those of THP-1 and THP-1PMA cells (b). MFI—mean fluorescence intensity, arbitrary units.
