# Supplementary material for: Macrophage-like THP-1 Cells Derived from High-Density Cell Culture Are Resistant to TRAIL-Induced Cell Death via Down-Regulation of Death-Receptors DR4 and DR5
Source: Biomolecules. 2022 Jan 18;12(2):150. doi: 10.3390/biom12020150 (PMC8961584; doi:10.3390/biom12020150)
Supplement: Supplementary file 1 [file biomolecules-12-00150-s001.zip › Supplement Tables.pdf]

Supplement Tables

Table S1. STR profile of THP-1 cells in comparison with the reference profile of THP-1 from ATCC.

Loci	Genotype of THP-1 cells	Closest match (THP-1 ATCC)
D5S818	11,12	11,12
D13S317	13,13	13,13
D7S820	10,10	10,10
D16S539	11,12	11,12
vWA	16,16	16,16
TH01	8,9,3	8,9,3
AMEL	X,Y	X,Y
TPOX	8,11	8,11
CSF1PO	11,13	11,13

Table S2. Comparison of STR profiles of THP-1ad and THP-1 cells for an expanded set of DNA loci compared to ATCC.

Loci	Genotype of THP-1ad cells	Genotype of THP-1 cells
**AMEL**	**X,X**	**X,Y**
**D3S1358**	**15,17**	**15,17**
**TH01**	**8,9,3**	**8,9,3**
**D12S391**	**19,19**	**19,19**
**D1S1656**	**16,16**	**16,17**
**D10S1248**	**14,14**	**14,14**
**D22S1045**	**11,15**	**11,15**
**D2S441**	**10,11**	**10,11**
**D7S820**	**10,10**	**10,10**
**D13S317**	**13,14**	**13,13**
**FGA**	**24,25**	**24,25**
**TPOX**	**8,11**	**8,11**
**D18S51**	**13,14**	**13,14**
**D16S539**	**11,12**	**11,12**
**D8S1179**	**10,14**	**10,14**
**CSF1PO**	**11,13**	**11,13**
**D5S818**	**11,12**	**11,12**
vWA	16,16	16,16
D21S11	30,31.2	30,31.2
SE33	20,30.2	20,30.2

Table S3. Specific chromosomal aberrations characteristic of THP-1ad cells, identified by the aCGH method.

chr	Chr. aberrations	Size (Mbps)	Aberrations
chr1	dup(1)p21.1p13.3	4,1	1p21.1p13.3(107,134,686-111,200,373)x10
	dup(1)p13.3p13.2	4,1	1p13.3p13.2(111,200,374-114,373,840)x5
	dup(1)p13.2p11.2	7,0	1p13.2p11.2(114,373,841-121,330,906) x10
	dup(1)q21.1	2,3	1q21.1(142,617,943-144,912,460)x3
	dup(1)q21.1	0,4	1q21.1(144,912,461-145,291,770)x4~5
	dup(1)q21.1q21.3	9,3	1q21.1q21.3(145,291,771-154,618,697)x3
	dup(1)q21.3q22	0,6	1q21.3q22(154,618,698-155,236,228)x4
	dup(1)q22	1,2	1q22(155,236,229-156,462,310)x3
	dup(1)q41q44	32,1	1q41q44(217,145,425-249,212,668)x3
chr3	del(3)p21.31	0,06	3p21.31(45,438,685-45,501,534)x1
chr5	dup(5)p15.33p13.3	32,1	5p15.33p13.3(22,149-32,143,878)x3
	dup(5)p13.3p12	13,9	5p13.3p12(32,143,879-46,022,523)x4
	dup(5)q21.3	0,5	5q21.3(106,188,878-106,617,966)x3
	dup(5)q21.3	0,3	5q21.3(107,396,307-107,700,282)x3
	dup(5)q21.3q34	53,7	5q21.3q34(108,255,922-161,908,453)x3
	dup(5)q34	0,3	5q34(161,908,454-162,186,029)x4
	dup(5)q34q35.3	18,5	5q34q35.3(162,186,030-180,676,122)x3
chr6	dup(6)q21	2,5	6q21(107,814,067-110,341,820)x8~9
chr7	dup(7)	159,3	(7)x3
chr8	dup(8)p23.2	2,2	8p23.2(3,689,391-5,935,998)x3
	dup(8)q22.2	0,3	8q22.2(100,160,197-100,508,951)x3
	dup(8)q22.2q24.3	45,4	8q22.2q24.3(100,856,029-146,280,020)x3
chr9	hmz(9)p21.2p13.2	11,3	9p21.2p13.2(25,660,228-36,977,914)x2 hmz
chr10	hmz(10)p15.3p13	13,2	10p15.3p13(307,807-13,494,876)x2 hmz
	dup(10)p13	0,4	10p13(13,418,583-13,778,564)x3
	dup(10)p13p11.21	23,9	10p13p11.21(13,797,823-37,638,396)x2 hmz
chr11	dup(11)q23.3q24.1	4,0	11q23.3q24.1(118,374,043-122,403,824)x3
	dup(11)q24.1	0,5	11q24.1(122,403,825-122,893,472)x4~5
	dup(11)q24.1	12,0	11q24.1(122,893,473-134,934,196)x3
chr17	dup(17)q21.31	0,2	17q21.31(44,476,634-44,694,283)x3
chr18	dup(18)p11.32	0,1	18p11.32(2,594,682-2,731,123)x3
chr20	dup(20)p11.23p11.21	1,8	20p11.23p11.21(20,809,316-22,628,267)x4
chrX	del(X)p22.33	2,5	Xp22.33(219,609-2,676,167)x1~0
chrY	del(Y)	57,2	Yx(0)

Table S4. Analyses of cytokine production by THP-1, THP-1ad, THP-1PMA and PBDM cells.

Cytokines	THP-1	THP-1ad	THP-1PMA	PBDM
IL-1β	0,22±0,07	0,33±0,05	71,10±4,62	0,52±0,08
IL-1ra	37,05±5,91	48,59±3,36	19605,53±1159,64	1183,00±152,85
IL-2	0,60±0,13	1,49±0,29	139,74±11,18	2,17±0,13
IL-4	0	0,05±0,01	1,78±0,1	0,1±0,02
IL-5	1,81±0,41	4,48±0,37	51,79±1,77	4,55±0,64
IL-6	0,34±0,01	0,63±0,07	424,40±48,25	0,70±0,06
IL-7	1,62±0,27	1,49±0,31	6,78±1,98	1,22±0,01
IL-8	1,61±0,28	9,36±0,61	23,59±1,96	2,67±0,37

IL-9	2,05±0,30	41,99±7,95	33284,00±0,01	1233,93±190,70
IL-10	2,01±0,37	2,17±0,18	7,29±0,91	2,13±0,47
IL-12p70	0,26±0,12	0,31±0,10	3,18±0,29	0,41±0,10
IL-13	0,37±0,07	0,49±0,05	1,28±0,08	0,44±0,05
IL-15	16,71±4,55	45,23±2,88	127,97±6,71	24,86±1,59
IL-17A	0,91±0,20	1,68±0,11	38,23±2,12	2,79±0,28
Eotaxin	0	0	3,14±0,22	0,08±0,02
Basic FGF	0	4,97±0,7	69,04±4,02	10,12±0,52
G-CSF	0	16,65±3,34	280,11±16,84	22,08±3,77
GM-CSF	0	0,34±0,01	6,54±0,22	0,44±0,09
IP-10	0	65,22±8,46	2077,67±179,09	97,09±4,18
MIP-1α	0	1,98±0,36	125,06±14,49	147,72±21,06
IFN-γ	0,56±0,10	1,47±0,44	48,31±5,46	25,59±1,48
MCP-1	0,50±0,15	44,68±6,29	27326,00±121,62	92,18±5,01
MIP-1β	2,14±0,25	39,54±3,03	6935,00±0,01	18,91±2,38
PDGF-BB	5,28±1,36	7,17±1,20	76,08±3,26	30,84±5,05
Rantes	40,59±5,26	1233,50±183,41	2805,50±299,49	54,56±7,59
TNF-α	2,57±0,01	27,78±1,61	8050,75±784,78	27,01±2,61
VEGF	27,33±4,97	70,03±4,89	532,75±41,00	51,97±9,45
