# Supplementary figures and images for: Macrophage-like THP-1 Cells Derived from High-Density Cell Culture Are Resistant to TRAIL-Induced Cell Death via Down-Regulation of Death-Receptors DR4 and DR5
Source: Biomolecules. 2022 Jan 18;12(2):150. doi: 10.3390/biom12020150 (PMC8961584; doi:10.3390/biom12020150)

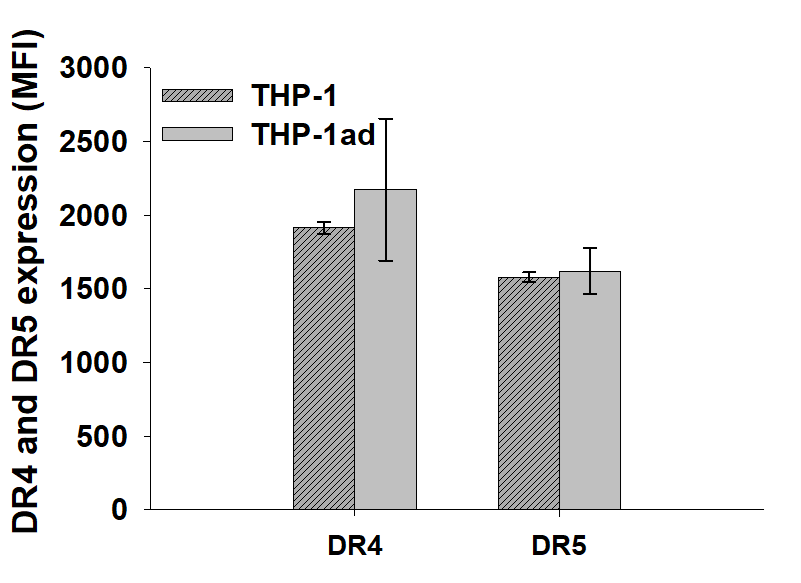

Supplement: Supplementary file 1 [file biomolecules-12-00150-s001.zip › Figure S5a.TIF]

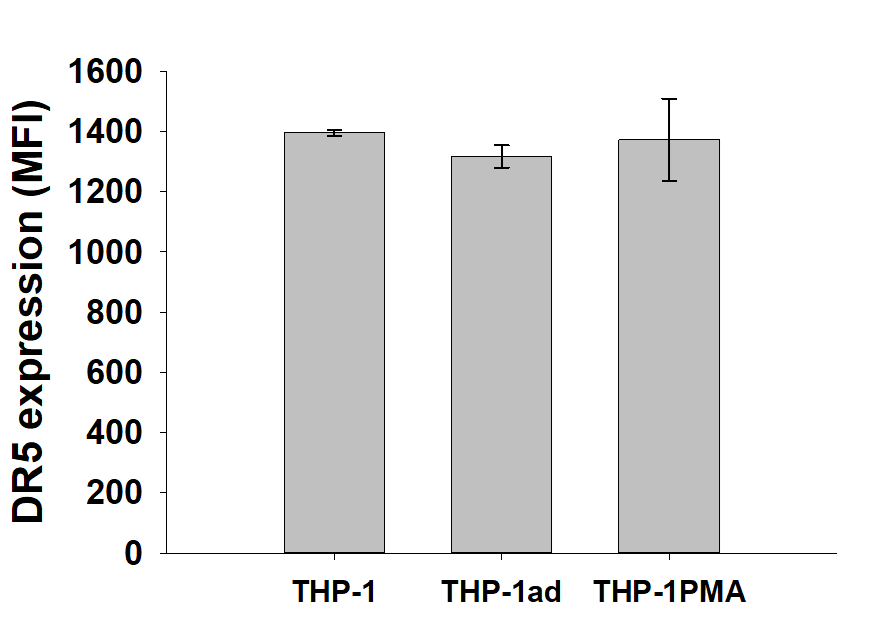

Supplement: Supplementary file 1 [file biomolecules-12-00150-s001.zip › Figure S5b.TIF]

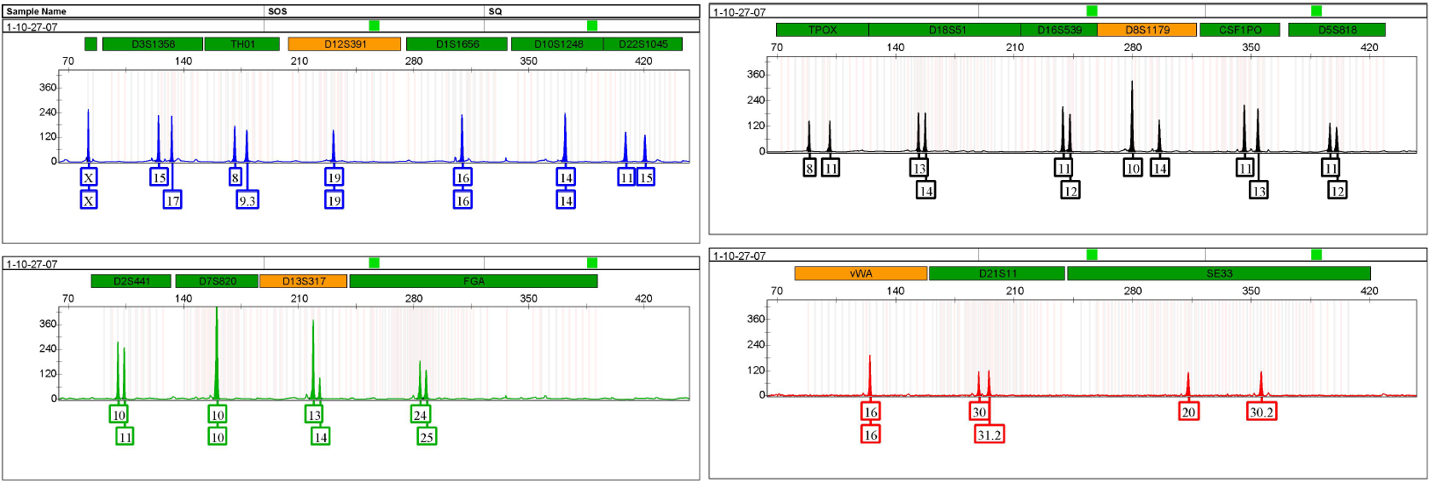

Supplement: Supplementary file 1 [file biomolecules-12-00150-s001.zip › S1a.tif]

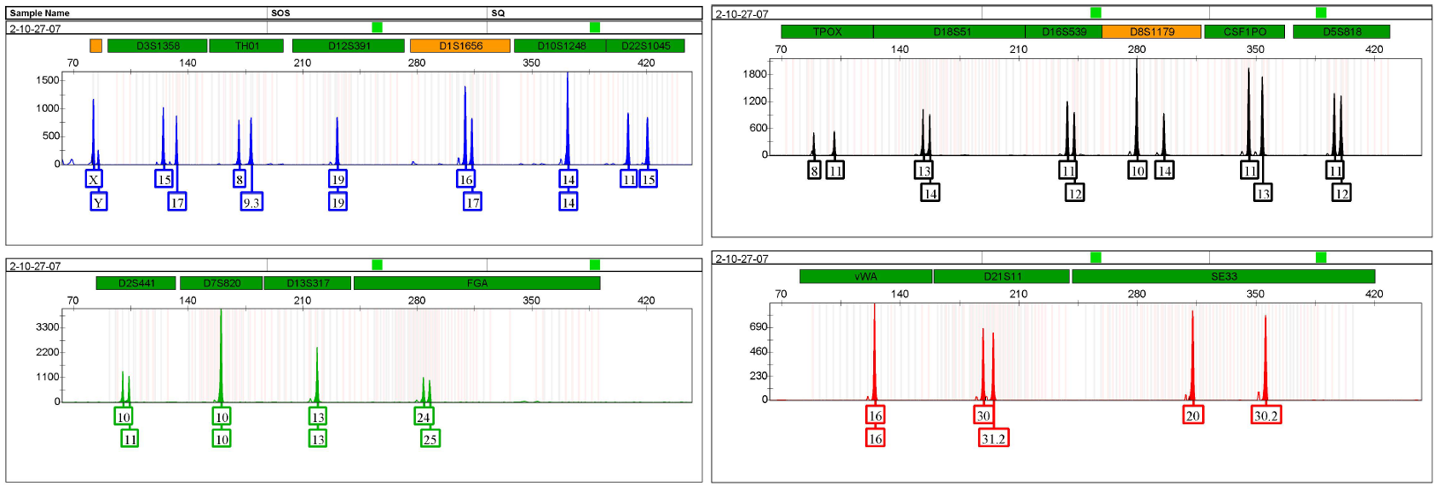

Supplement: Supplementary file 1 [file biomolecules-12-00150-s001.zip › S1b.tif]

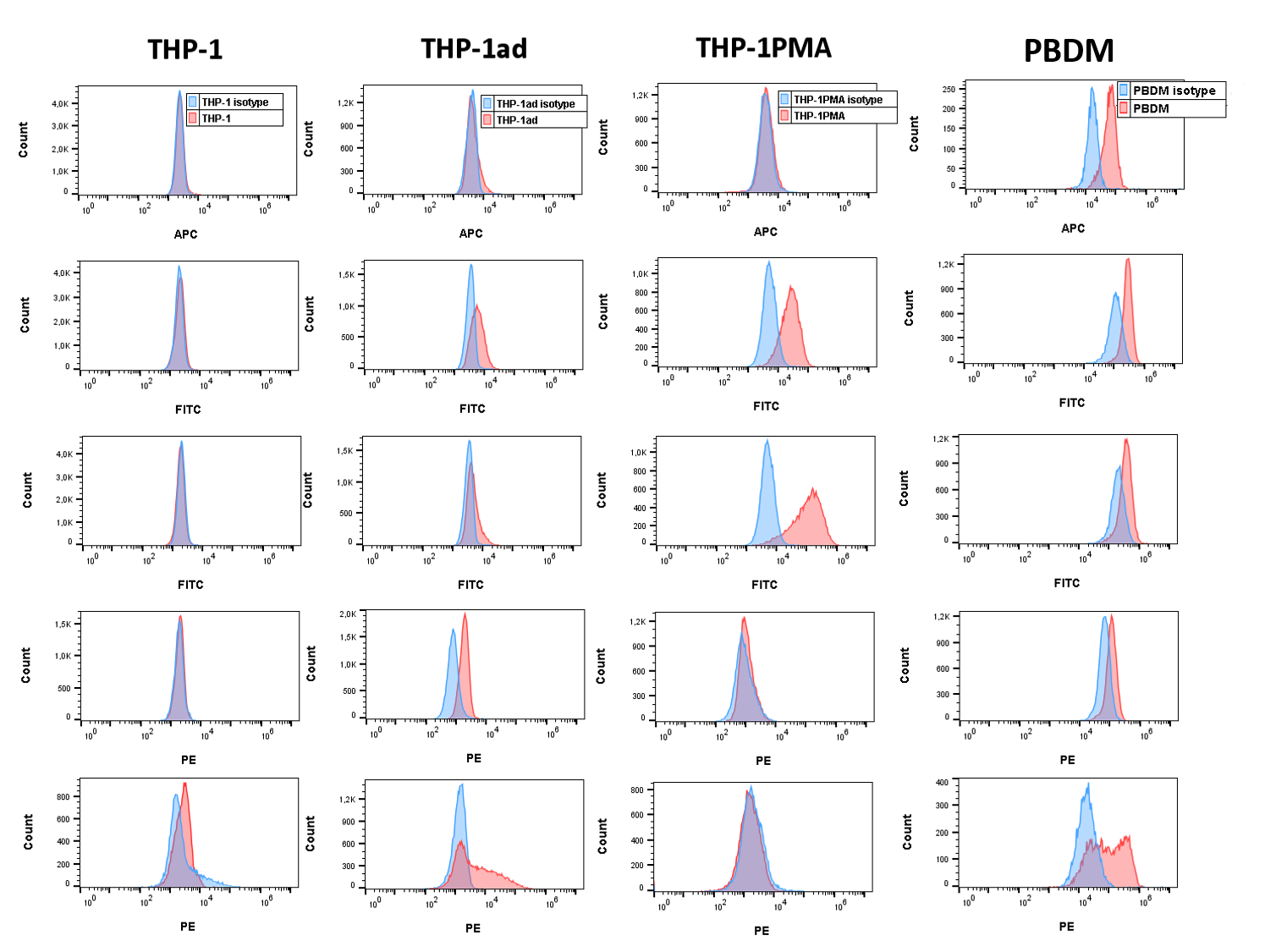

Supplement: Supplementary file 1 [file biomolecules-12-00150-s001.zip › S2.tif]

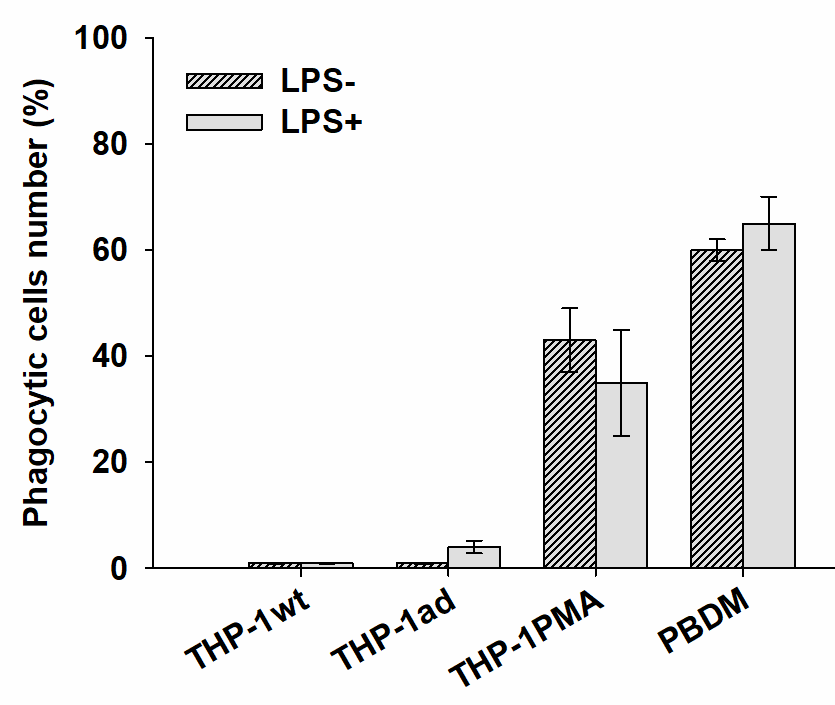

Supplement: Supplementary file 1 [file biomolecules-12-00150-s001.zip › S3a.tif]

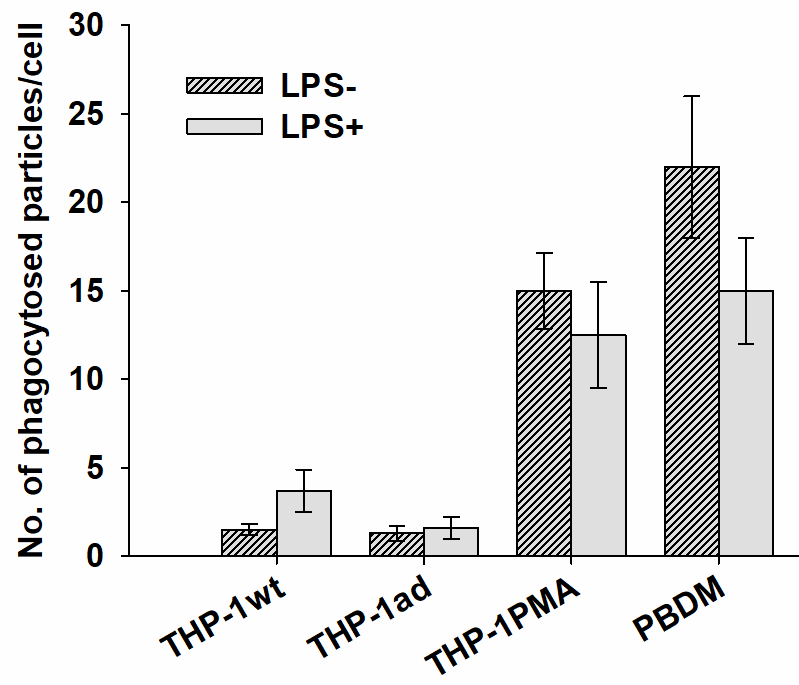

Supplement: Supplementary file 1 [file biomolecules-12-00150-s001.zip › S3b.tif]

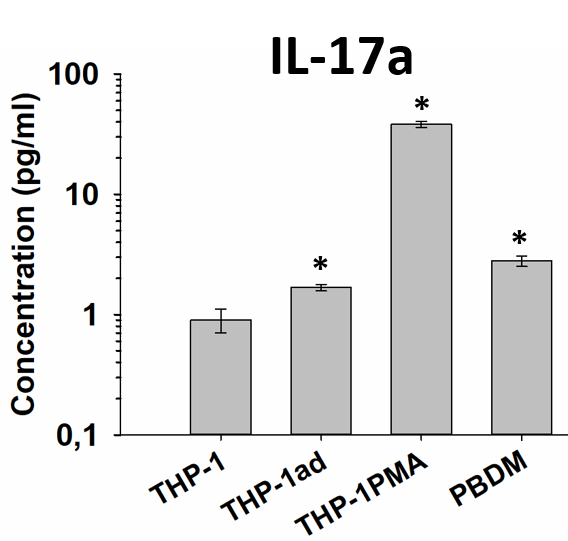

Supplement: Supplementary file 1 [file biomolecules-12-00150-s001.zip › S4(IL-17a).tif]

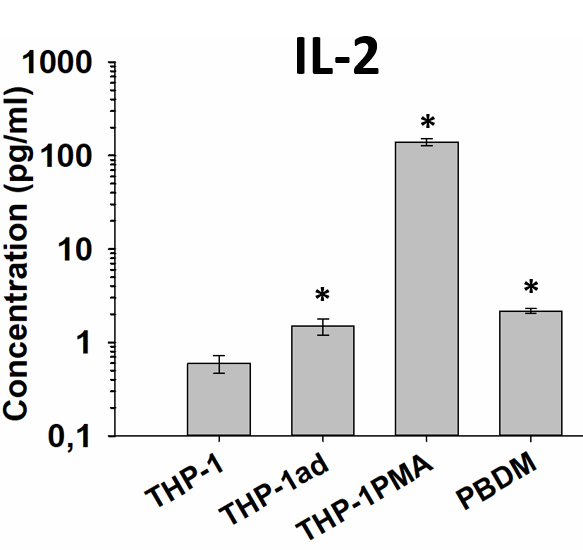

Supplement: Supplementary file 1 [file biomolecules-12-00150-s001.zip › S4(IL-2).tif]

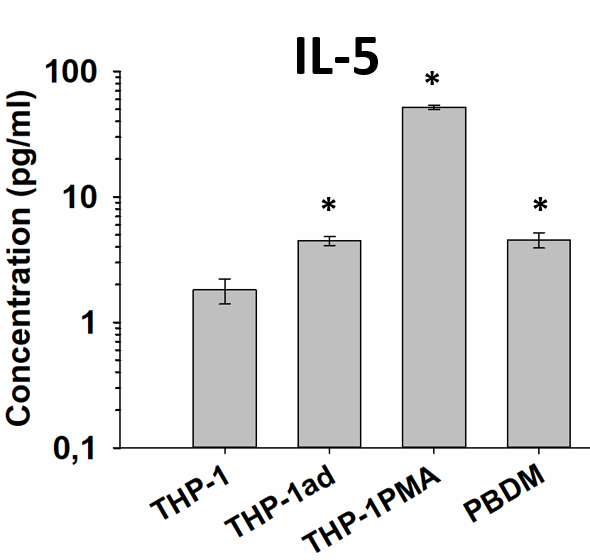

Supplement: Supplementary file 1 [file biomolecules-12-00150-s001.zip › S4(IL-5).tif]

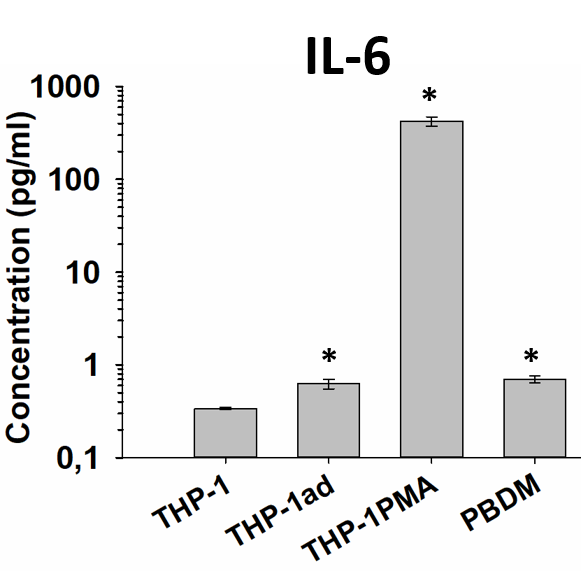

Supplement: Supplementary file 1 [file biomolecules-12-00150-s001.zip › S4(IL-6).tif]

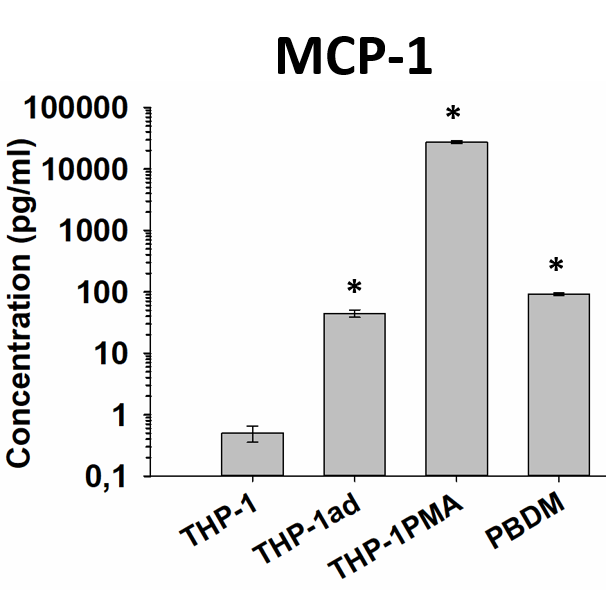

Supplement: Supplementary file 1 [file biomolecules-12-00150-s001.zip › S4(MCP-1).tif]

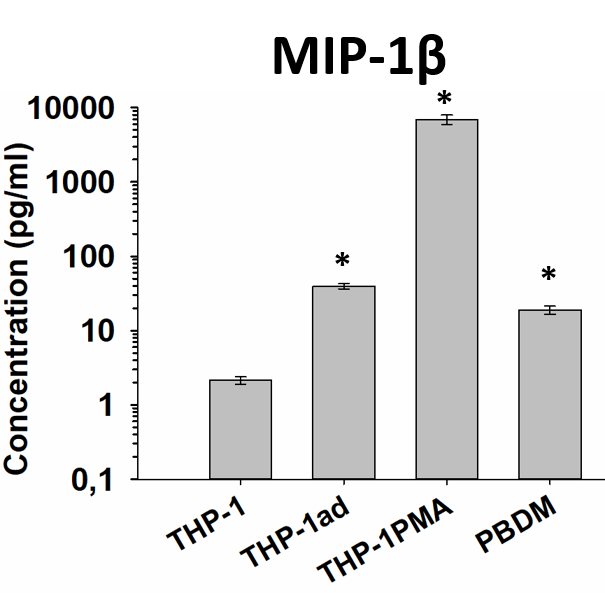

Supplement: Supplementary file 1 [file biomolecules-12-00150-s001.zip › S4(MIP-1b).tif]

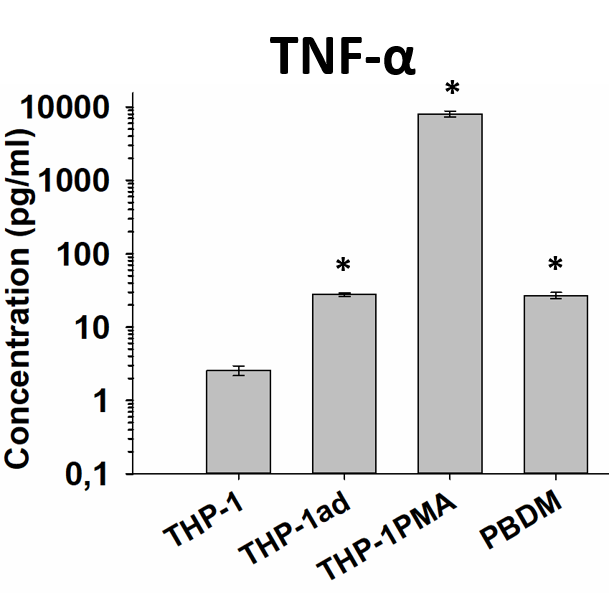

Supplement: Supplementary file 1 [file biomolecules-12-00150-s001.zip › S4(TNF-a).tif]

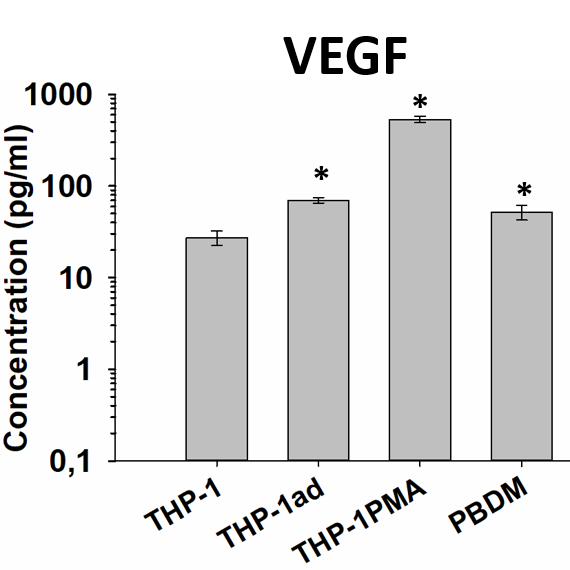

Supplement: Supplementary file 1 [file biomolecules-12-00150-s001.zip › S4(VEGF).tif]
